# Mesopore-Rich Activated Carbons for Electrical Double-Layer Capacitors by Optimal Activation Condition

**DOI:** 10.3390/nano9040608

**Published:** 2019-04-12

**Authors:** Hye-Min Lee, Kay-Hyeok An, Soo-Jin Park, Byung-Joo Kim

**Affiliations:** 1 Research Center for Carbon Convergence Materials, Korea Institute of Carbon Convergence Technology, Jeonju 54852, Korea; hmlee2014@hanmail.net; 2 Department of Chemistry, Inha University, Incheon 22212, Korea; 3 Department of Nano & Advanced Materials Engineering, Jeonju University, Jeonju 55069, Korea

**Keywords:** hard carbon, steam activation, polymeric precursor, electrical double-layer capacitor

## Abstract

In this study, activated polymer-based hard carbon using steam activation (APHS) with mesopore-rich pore structures were prepared for application as electrodes in electrical double-layer capacitors (EDLC). The surface morphologies and structural characteristics of APHS were observed using scanning electron microscopy and X-ray diffraction analysis, respectively. The textural properties were described using Brunauer-Emmett-Teller and Barrett-Joyner-Halenda equations with N_2_/77 K adsorption isotherms. APHS were prepared under various steam activation conditions to find optimal ones, which were then applied as electrode materials for the EDLC. The observed specific surface areas and total pore volumes of the APHS were in the range 1170–2410 m^2^/g and 0.48–1.22 cm^3^/g, respectively. It was observed that pore size distribution mainly depended on the activation time and temperature, and that the volume of pores with size of 1.5–2.5 nm was found to be a key factor determining the electrochemical capacity.

## 1. Introduction

Electrical double-layer capacitors (EDLCs) are very attractive for use in potential energy storage devices because of their high power density, quick charge-discharge rate, and long maintenance-free operation life [1,2,3]. Energy storage behavior of an EDLC arises mainly from the separation of electronic and ionic charges at the interface between the electrode materials and the electrolyte solution [4]. Therefore, the electrochemical behavior of an EDLC is determined by the textural properties of the active material. Activated carbons (AC) have excellent textural properties and high electrical conductivity, which have made them the most widely used active materials for EDLCs [5,6,7].

Many studies have focused on the specific surface area among the textural properties of AC [5,6]; however, Baek’s research showed that different pore structures are also required, depending on the salt and solvent of the electrolyte [7]. In conclusion, to improve the electrochemical properties of EDLCs, it is necessary to optimize the pore structure of the AC for each electrolyte.

The pore structure of AC is known to be greatly influenced by the precursor [8,9,10], activation method [11,12,13,14], and pyrolysis conditions [15,16]. The final pore size distribution is mainly determined by the activation method. ACs are prepared by physical activation (gasification of a char in oxidizing gases) or by chemical activation (pyrolysis of precursor impregnated with chemical reagents).

Chemical activation is generally done by mixing carbonaceous materials with chemical activating agents (KOH, H_3_PO_4_, ZnCl_2_, etc.), followed by pyrolysis at 400–900 °C [17,18,19]. This process gives rise to AC with a high specific surface area (>2000 m^2^/g) mainly of micropores, with some sub-mesopores [5,17]. Such a high specific surface area is ascribed to partial gasification and expansion of the interlayer spacing between crystallites through simultaneous intercalation and deintercalation [17]. However, this approach has disadvantages that include corrosiveness of the chemical agents and the washing process that is necessary to remove the chemical agents.

Physical activation is done by carbonization of carbon precursors in an inert atmosphere to remove non-carbon elements, followed by activation in the presence of suitable oxidizing gasifying agents (O_2_, CO_2_, or H_2_O) to develop the porosity, usually in the temperature range 600–1200 °C [14,15,16]. Pores are formed by the oxidation of crystallites by physical activation, during which the size of the crystallite affects pore characteristics as much as the activation method does [16]. Baek et al., reported that hard carbon (HC) of low crystallinity could be used to obtain AC of high specific surface area using physical activation [14,15].

In general, the textural properties (specific surface area, pore volume) of AC resulting from chemical activation are known to be better than those from physical activation [17]. This is why most AC studies for EDLC focus on chemical activation methods. However, the process cost of chemical activation is much higher than that of physical activation. Thus, research is needed to produce AC with excellent pore characteristics through physical activation.

Most commercial grade AC is derived from naturally occurring precursors such as wood [19], coal [20], and coconut shells [21]. However, naturally occurring precursors contain large quantities of ash [22]. For the production of AC, ash causes problems such as capacity reduction, gas creation, and swelling of the EDLC [23,24]. Therefore, AC produced from naturally occurring precursors requires a separate ash removal process.

Polymeric precursors have structural features similar to those in coal, but contain many fewer mineral impurities (from catalysts), which can be controlled to very low levels during their synthesis [25]. However, polymer-based AC with high specific surface area has been reported as a result of chemical activation [17,24]. Generally, a polymer-based precursor with high carbonization yield has high crystallinity; therefore, it is difficult to produce AC having a high specific surface area by physical activation [26].

In this work, activated polymer-based hard carbon using steam activation (APHS) with a high specific surface area and mesopore-rich pore structure was prepared from polymeric precursors with low crystallinity. The pore structure of the APHS obtained was studied using N_2_ adsorption. The APHS thus prepared was applied as electrodes for the EDLC, and its specific capacitance was discussed in relation to the pore structure.

## 2. Experiment Details

### 2.1. Materials

Polymeric precursor (polyurethane) was obtained from Aekyung Petrochemical Co., Ltd. (Wanju, Korea) [27]. Portions of the polymeric precursor (5 g) were heated to 900 °C at 10 °C/min in a self-made cylindrical steel tube furnace (SiC heater: length 1000 mm, diameter 100 mm) under N_2_ gas (99.999%) and kept at a target temperature for 1 h to obtain 2 g of carbonized polymer-based HC.

### 2.2. Steam Activation Optimizaiton

Activation of the HC was performed using steam as the physical reagent. Steam activation was performed in the same steel tube used for carbonization. The HC was heated (10 °C/min) to the required activation temperature (900 or 1000 °C) under N_2_ (300 mL/min), before the N_2_ was replaced with H_2_O (0.5 mL/min, liquid) with various activation times. The sample was then allowed to cool under N_2_ (300 mL/min). The samples were named as activated polymer-based hard carbons (APHS) and named APHS-activation temperature-activation time sequence according to activation conditions. The prepared AC was pulverized to 10 μm size using a ball mill (Pulverisette 23, Idar-Oberstein, Germany).

### 2.3. Characterization

The N_2_ adsorption isotherms of APHS were measured with a BELSORP-max (BEL JAPAN, Toyonaka, Japan) at liquid nitrogen temperature. For pore analysis, all samples were degassed at 573 K for 6 h with the residual pressure maintained at 10^−3^ torr, or less. The specific surface area was calculated for the relative pressure interval of 0.03–0.19 using the Brunauer-Emmett-Teller (BET) equation. [28] The total pore volume, V_Total_, was calculated from N_2_ adsorption data as the volume of liquid N_2_ at a relative pressure of 0.99. The mesopore volume, V_Meso_, was determined by the Barrett-Joyner-Halenda (BJH) method, and the micropore volume, V_Micro_, was obtained by subtraction of the mesopore volume from the total pore volume. Micropore and mesopore size and distribution were calculated using the non-local density functional theory (NLDFT) [29] and BJH method [30], respectively. The microstructure of the APHS was determined using an X-ray diffractometer (XRD, X’Pert Pro Diffractometer, PANalytical, Almelo, The Netherlands), employing a Rigaku SmartLab X-ray diffractor with a customized auto-mount and a Cu Kα (λ = 1.5406 Å) radiation source. Diffraction patterns were collected within the diffraction angles from 5° to 90° at a rate of 2°/min. The interlayer spacing (d_002_, d_10*l*_) of the samples were calculated using Bragg’s Law (2dsinθ = nλ) to the position of the (002) and (10*l*) peak, respectively. The size of crystalline under the Scherrer equation [31] can be expressed as follows:

(1) $$L=\frac{K\lambda }{\mathrm{Bcos}\theta }$$

In this equation, the constant K is 0.9 and 1.84 when calculated from crystalline height (L_c_) and crystalline diameter (L_a_), respectively. λ is the wavelength of the X-ray, and B is the full width at half maximum (FWHM) calculated from the radian.

### 2.4. Electrochemical Measurements

The slurry was prepared by mixing 80 wt% active materials (activated HC, APHS), 10 wt% conductive agents (carbon black, Super P, TIMCAL, Bodio, Switzerland), 10 wt% binder (carboxymethyl cellulose and styrene-butadiene rubber) dispersed in water. The slurries were coated on the aluminum foil using a doctor blade technique. The thickness of the coating layer was controlled to 127 μm. The coated foils were then first dried in an oven at 80 °C for over one hour. To remove any remaining water, the electrodes were further dried in a vacuum oven at 120 °C overnight. After the drying was completed, the electrodes were roll pressed to a thickness of less than 80 μm using a two-roll press at 80 °C.

For electrochemical testing, a CR2032 coin cell consisting of two punched electrodes 12 mm in diameter, punched cellulose paper separators 16 mm in diameter (NKK, Kochi, Japan), and 1M (C_2_H_5_)_4_NBF_4_/propylene carbonate (1M TEABF_4_/PC) were used. All cell assembly was carried out in a dry room where the dew point was below 60.0 °C.

The fifth cycle of galvanostatic charge/discharge (GCD) and the second cycle of cyclic voltammetry (CV) were used to evaluate the electrochemical performance of the samples. The GCD was charged at 10 mA and then discharged at 2 mA (0.1–2.4 V) for the coin cell using a charge/discharge tester (Maccor 4300 K, Maccor, Tulsa, OK, USA). CV and impedance spectrometry (EIS) were analyzed using a potentiostat (Bio-Logic VSP, Bio-Logic Science Instruments, Seyssinet-Pariset, France). CV studies were performed in the same potential range of GCD at a scan rate of 30 mV/s. The impedance Nyquist plots were recorded in the frequency range 10 mHz to 300 kHz. The cells produced were measured based on the capacitance per unit weight, and calculated only using the weight of active materials (F/g). The specific capacitance was calculated according to the GCD based on the following equation:

C_g_ = iΔt/mΔV (2)

where i is the discharge current (A), Δt is the discharge time (s), m is the mass of the electrode (g), and ΔV is the potential difference (V).

## 3. Results

### 3.1. X-ray Diffraction Analysis

**Figure 1:**
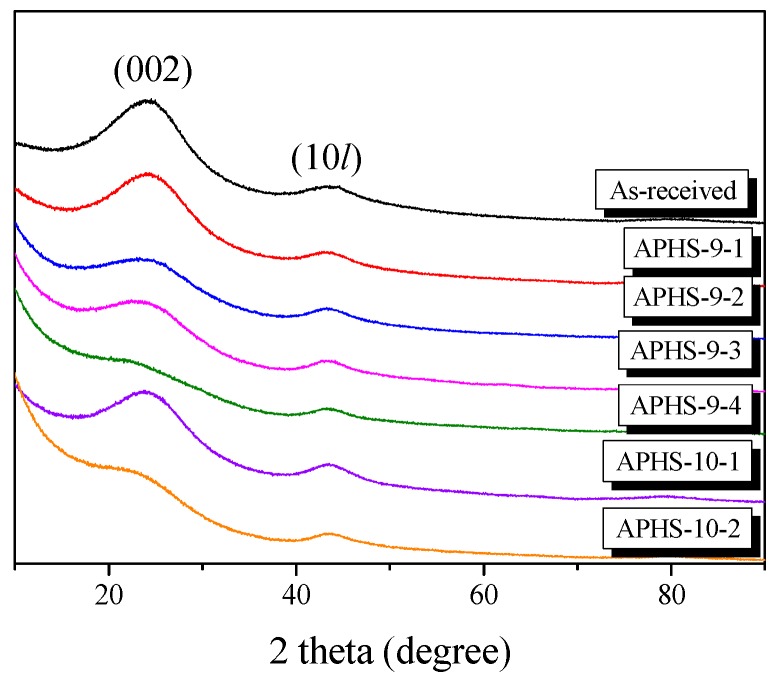
X-ray diffraction patterns of activated polymer-based hard carbon under various steam activation conditions.

**Figure 2:**
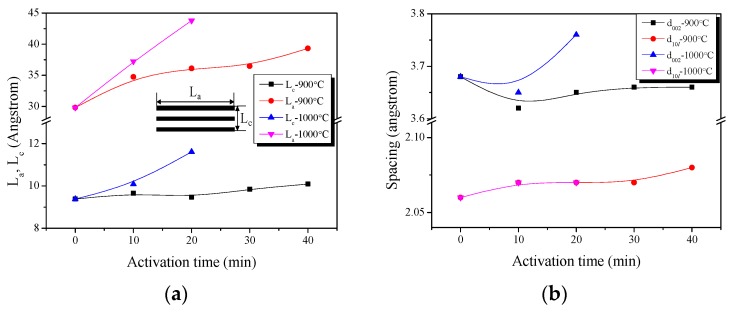
Structural characteristics of the activated polymer-based hard carbon as a function of various steam activation conditions: (**a**) structural parameters; (**b**) interplanar distance.

It is well known that XRD is a powerful technique for revealing detailed and precise microstructure, such as the interlayer spacing (d_002_) of AC, which is composed of thin layers with the same atomic positions as graphite within the layers. Figure 1 shows the X-ray diffraction profiles of the APHS prepared under different activation conditions. The APHS exhibits very broad diffraction peaks and the absence of a sharp peak reveals a predominant HC structure. The XRD pattern of the AC was very similar to that of the HC. In this work, the interlayer spacing of the APHS is about 3.62 to 3.76 Å, and that of the HC is 3.68 Å, as seen in Figure 2.

The oxidation behaviors of HC tend to operate in the amorphous regions and graphite edges [11]. It is widely known that amorphous carbon can be more easily oxidized than crystalline carbon can during the activation reactions [11,12,13]. If carbon atoms in the amorphous region of HC were removed first, the overall crystallinity of the HC could be increased due to decrease in a portion of the amorphous region, resulting in the increase of L_c_ or L_a_ in the final APHS.

As shown in Figure 2, L_c_ does not exhibit a large change at an activation temperature of 900 °C, but does gradually increase with the activation time. However, L_a_ increases steadily with increasing activation time. In particular, the largest increase is found for the sample that was activated for 10 min, followed by 30 to 40 min. It is believed that much oxidation of amorphous parts or small crystallites occurred in this section. This was confirmed in the yield of Table 1. Both L_c_ and L_a_ increase significantly at the activation temperature of 1000 °C. These results suggest that the activation reaction at high temperature is strong and rapid.

The interlayer spacing exhibited a large change at d_002_ and a small change at d_10*l*_. The interlayer spacing decreased up to APHS-9-1, and then increased. The increase in the interplanar distance d_002_ was clearly observed with increase in the activation time. More specifically, the distance decreased from 3.68 Å for the HC to 3.62 Å for APHS-9-1. The amorphous oxidation within HC is presumed to cause this decrease in the interplanar distance of d_002_. In addition, a similar trend is observed in APHS-10-1. After amorphous oxidation, the small crystallite is oxidized and increases the interplanar distance of d_002_. According to the activation progression, the specific surface area of APHS was increased by oxidation of small crystallites. Based on these observations, it can be postulated that long activation time and high temperature burn off the less ordered components and small crystallites, resulting in the increased specific surface area of the APHS.

### 3.2. Adsorption Isotherm and Textural Properties

**Figure 3:**
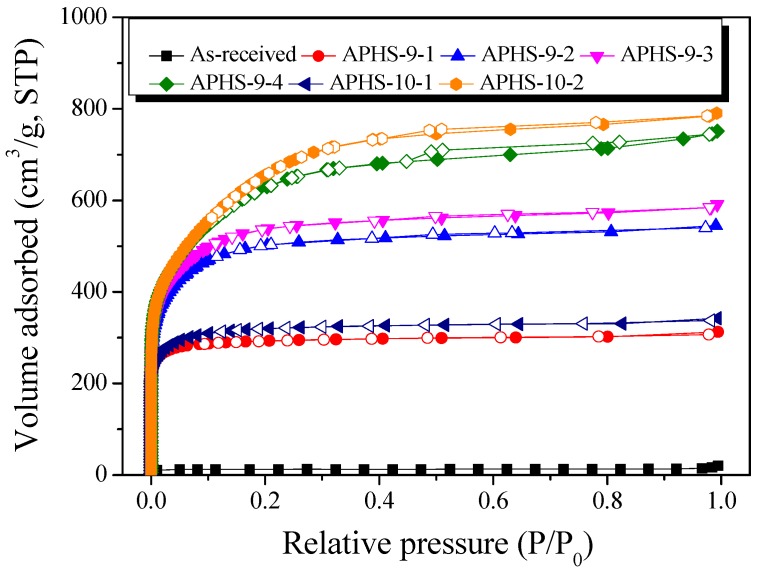
N_2_/77 K adsorption-desorption isotherm curves of activated polymer-based hard carbon under various steam activation conditions.

The adsorption isotherms of N_2_ at 77 K contain a large amount of information related to pore-structure. Because changes in the isotherms imply an alteration of pore structures; their measurements are the basis upon which to estimate the pore structure parameters. The specific surface area and pore structures of the APHS before and after activation were determined using N_2_ adsorption/desorption isotherms (Figure 3). There are six kinds of adsorption isotherm patterns according to the International Union of Pure and Applied Chemistry (IUPAC) classification [32], and each is indicated by a distinct pore structure. According to the IUPAC standards, the curves in Figure 3 all have Type I shape, largely consisting of micropores; longer activation time was associated with increased specific surface area and quantity of mesopores.

When the hysteresis curves were examined, hysteresis curves were rarely observed except for APHS-9-4 and APHS-10-2. This seems to be because the mesopores of these two samples were well-developed, so that many pores underwent interior development so that the shape of such pores changed to the shape of a jar.

The effect of the steam activation conditions on the specific surface area, total pore volume, and micropore volume are given in Table 1. The specific surface area and total pore volume increase with increasing activation temperature and time. As activation time and temperature increase during the steam activation process, the specific surface area increases from 50 to 2410 m^2^/g, and the total pore volume increases from 0.03 to 1.22 cm^3^/g.

When the pore characteristics were examined in detail at the activation temperature of 900 °C, APHS activated for 10 min developed only micropores. At up to 40 min of activation, both micropores and mesopores increased, with the former increasing from 0.02 to 0.78 cm^3^/g and the latter increasing from 0.01 to 0.38 cm^3^/g. In the case of APHS activated for 40 min, the volume of micropores increased from 0.74 to 0.78 cm^3^/g, while that of mesopores increased from 0.17 to 0.38 cm^3^/g. Because the activation yield was greatly reduced, mainly mesopores were increased. This is because micropores collapsed and developed into mesopores due to the activation reaction induced by steam oxidation. However, as the volume of micropores continued to increase, it was recognized that new micropores continued to be formed as the activation time increased. Activation at 1000 °C was observed to develop pores more quickly than activation at 900 °C. APHS-10-2 sample exhibited the highest specific surface area and total pore volume.

**Figure 4:**
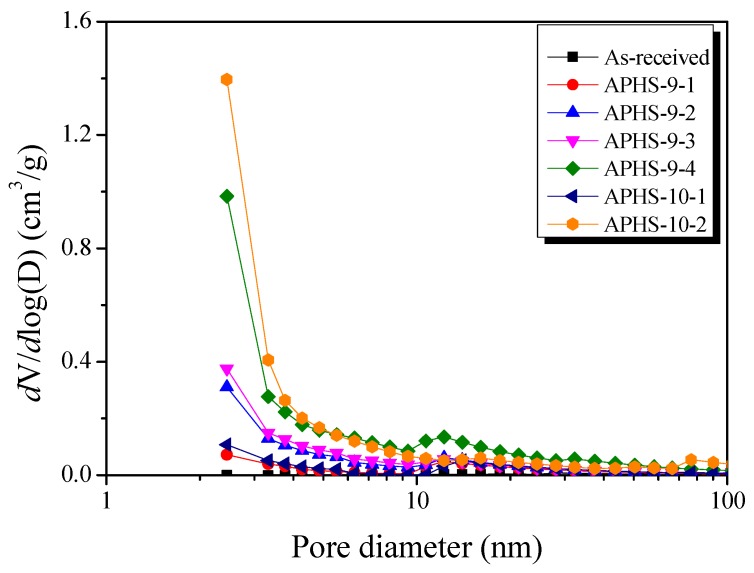
Pore size distribution of polymer-based hard carbon activated under various steam activation conditions using the BJH method.

Figure 4 exhibits the mesopore size distributions after application of the BJH method. All samples have their highest intensity peak around 2.5 nm pore diameter. APHS-9-1 and APHS-10-1, and APHS-9-4 and APHS-10-2, were produced using different activation processes, but have similar specific surface areas. However, the APHS produced at higher temperatures exhibits greater mesopore volume. That is, the high activation temperature produced APHS that was richer in mesopores.

**Figure 5:**
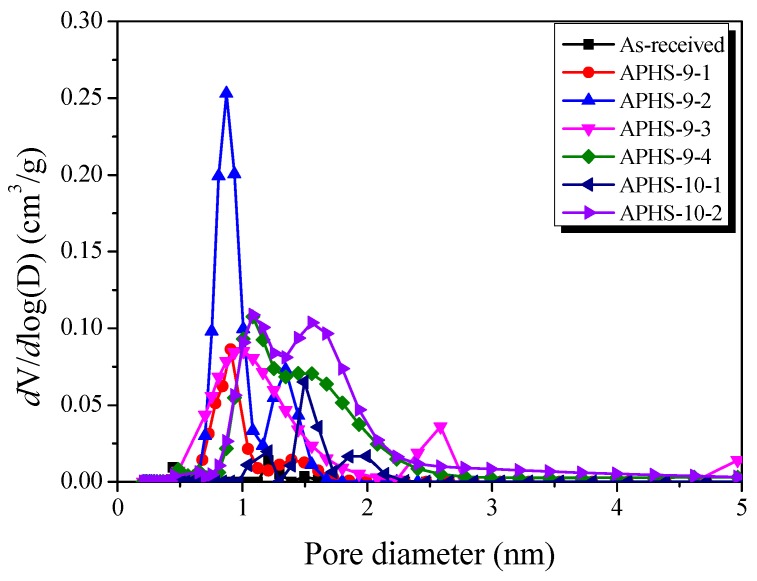
Pore size distribution of polymer-based hard carbon activated under various steam activation conditions using the non-local density functional theory method.

Figure 5 exhibits the micropore size distributions created by the NLDFT method. As activation time increases, narrow micropores develop. High micropore volume is observed from up to 20 min of activation time in the diameter range from 0.75 to 1.0 nm. When activation time exceeds 20 min and stretches to 30 min, micropore volume decreases in the diameter range from 0.75 to 1.0 nm, while the micropore volume increases in the diameter range from 1.0 nm and more. Moreover, the pore size distribution becomes broader.

It is widely known that steam activation is a process that develops pores by oxidizing the carbon atoms of the precursors [13,16]. During the initial increase of activation time, micropores with small pore diameters are formed first as a result of the reaction between steam and carbon. With increasing activation time, the oxidation of crystallites increases, leading to increased specific surface areas. However, with additional activation time, the pores developed in the initial state start to deepen, enlarge, and perhaps merge, resulting in the observed increase in the average pore width. In addition, new micropores develop within the mesopores, and the specific surface area continues to increase.

### 3.3. Electrochemical Characterization

The electrochemical properties, including the galvanostatic discharge curves of the APHS electrodes, were studied using an electrolyte of 1M TEABF_4_/PC. Theoretically, the capacitance of the AC is proportional to its specific surface area [6]. However, only the surface of the pores that the ions can access can contribute to double layer capacitance [5,7]. In particular, the pore size distribution has been considered the most important parameter, because the accessibility of ion molecules in an electrolyte strongly depends on the pore size of the electrodes. The sizes of non-solvated ions and the sizes of solvated ions in 1M TEABF_4_/PC is 0.34 to 1.40 nm [7]. Therefore, mesopores are more useful than micropores for EDLCs, especially for non-aqueous and ionic liquid (IL) EDLCs with larger ions. Baek et al., reported a close relationship between specific capacitance and the pore size of activated carbon (2–5 nm and >5 nm) in 1M TEABF_4_/PC [7].

**Figure 6:**
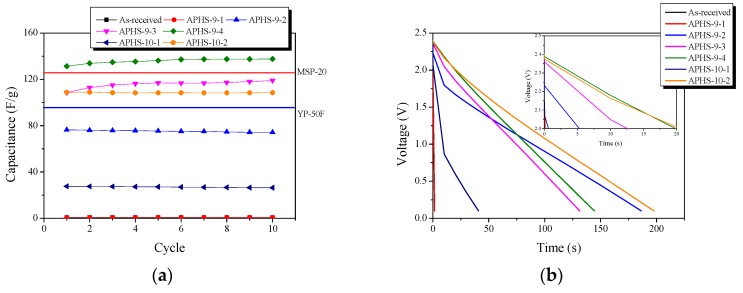

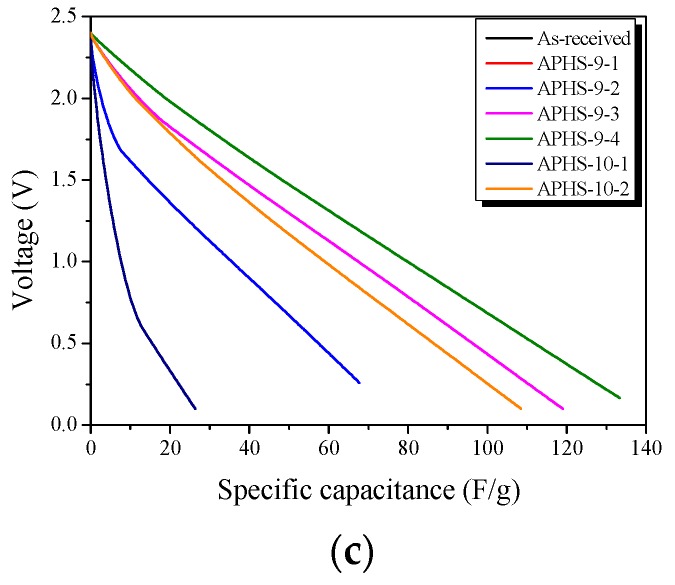
Electrochemical performance of the activated polymer-based hard carbon as a function of a cycle under various steam activation conditions: (**a**) initial specific capacitance; (**b**) discharge curves; (**c**) specific capacitance curves. Because the discharge data was not converted to the value per weight of electrode material, they cannot be compared with absolute value.

Figure 6a exhibits a change in the specific capacitance according to the charge/discharge cycle. All APHS exhibit stable initial specific capacitance. The specific capacitance is estimated from the galvanostatic charge/discharge curve (Figure 6b), which corresponds to the calculated specific capacitance of the electrode. The specific capacitance of all the APHS samples increases with increasing activation times and temperature. APHS-9-1 has very low capacity due to small pore size and low pore volume. The mobility of the ions within the pores is greatly influenced by pore size. If the pores are too small to allow easy access to electrolyte ions, they will not contribute to double-layer capacitance. APHS-9-2 has specific capacitance higher than that of APHS-9-1 because micropores and mesopores were significantly increased by activation. APHS-9-3 exhibits micropore and mesopore volume slightly increased over those of APHS-9-2; however, the specific capacitance increases significantly from 73.2 to 115.8 F/g. As discussed in Figure 5, the main pore diameter is distinctively enlarged due to expansion of the previously generated micropores and to collapse of micropore walls. Therefore, it is considered that the adsorption capacity of ions would be increased to provide easy access to electrolyte ions by the enlarged pores. In this work, APHS-9-4 exhibits the best mesopore fraction and 136.1 F/g of energy storage ability. The galvanostatic discharge curve of APHS-9-4 exhibited a straight line, typical of EDLC, with a non-IR drop. (Because the discharge data was not converted to the value per weight of the electrode material, they cannot be compared with the absolute value.) This is about 148% and 108% higher than the YP50F (95 F/g, coconut shell origin, physical activation with steam, Kuraray Chemical Co., LTD., Osaka, Amagasaki, Japan) and MSP20 (125 F/g, phenol resin origin, chemical activation with KOH, Kansai Coke and Chemicals, Japan) electrode capacity, respectively [16,33]. The porosity of APHS-9-1 and APHS-10-1 are very similar, but the specific capacitance of APHS-10-1 is higher than that of APHS-9-1. In Figure 5, the pore diameter of APHS-10-1 is larger than that of APHS-9-1, with easy access to electrolyte ions. APHS-10-2 has the highest specific surface area and mesopore volume. However, APHS-10-2 exhibits lower specific capacitance than APHS-9-3 and APHS-9-4. As seen in Figure 5, the pore width of APHS-10-2 is wider than that of APHS-9-3 and APHS-9-4. Therefore, APHS-10-2 has low capacitance despite the fact that APHS-10-2 has the best pore properties.

**Figure 7:**
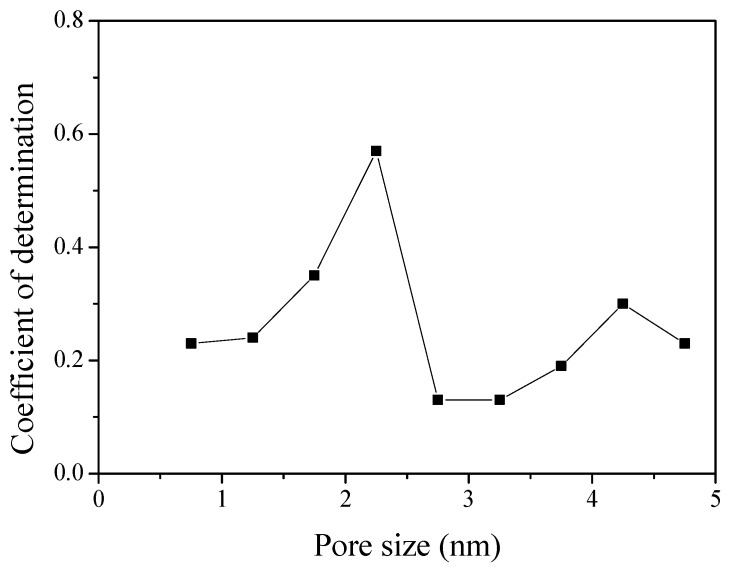
Correlation between the specific capacitance of activated polymer-based hard carbon with various pore volume. The *X*-axis exhibits the average pore size distribution. It was plotted using the pore volume according to the pore diameter in 0.5 nm units and the average value of each pore size distribution.

Figure 7 exhibits the result of plotting the pore volume according to pore diameter in 0.5 nm units using NLDFT, and then plotting the coefficient of determination with specific capacitance. An empirical linear fit was used to evaluate the contributions from each divided pore volume. The *X*-axis is exhibited using the average value of each pore size distribution. The R^2^ coefficient of determination (R^2^ = 1 − SS_res_/SS_tot_, SS_res_ is residual sum of squares and SS_tot_ is total sum of squares) exhibits a trend of increase and then decrease again after the occurrence of the highest value in the results (1.5–2.5 nm pore diameter).

**Figure 8:**
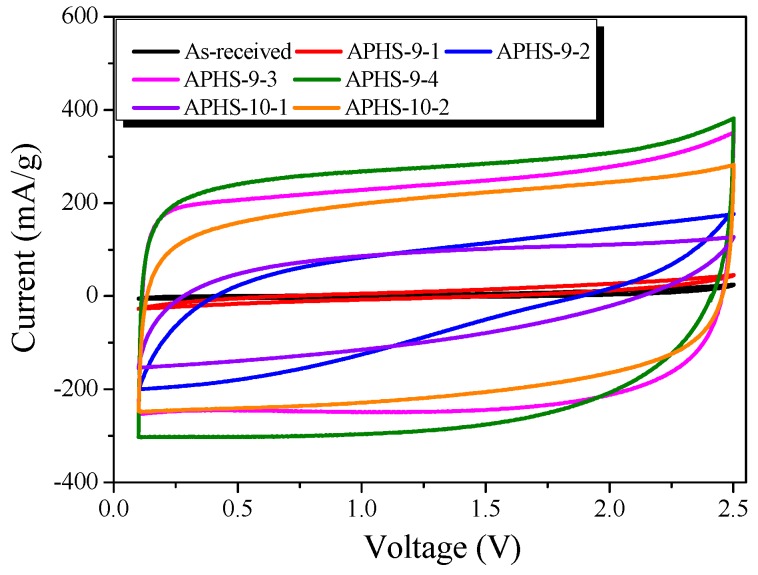
Cyclic-voltammetry curves of polymer-based hard carbon activated under various steam activation conditions.

The typical cyclic voltammograms (CV) of the capacitor cells are shown in Figure 8. All the CV curves are rectangular without obvious redox current on both positive and negative sweeps over the whole potential range of 0.1 to 2.5 V at 30 mV/s in the organic electrolyte (1M TEABF_4_/PC). At longer activation time, CV curves gradually change into rectangles. Moreover, APHS-9-3 and APHS-9-4 show a symmetric, quasi-rectangular shape profile typical of ideal EDLCs. This is consistent with the results of pore characteristic analysis and seems to be because the pores are better developed than those of other ACs. Generally, the specific capacitance of an electrode is in proportion to the integrated area of its CV profile under the same scan rate and voltage window (i.e., the larger the integrated area is, the greater the specific capacitance). As activation time increases, the area of the CVs tends to increase. The capacitance of the electrodes calculated from the CV curves decreases in the following order: APHS-9-4 > APHS-9-3 > APHS-10-2 > APHS-10-1 > APHS-9-2 > APHS-9-1. Among the carbon samples, APHS-9-4 shows the highest specific capacitance due to its uniform mesopores and high specific surface area.

**Figure 9:**
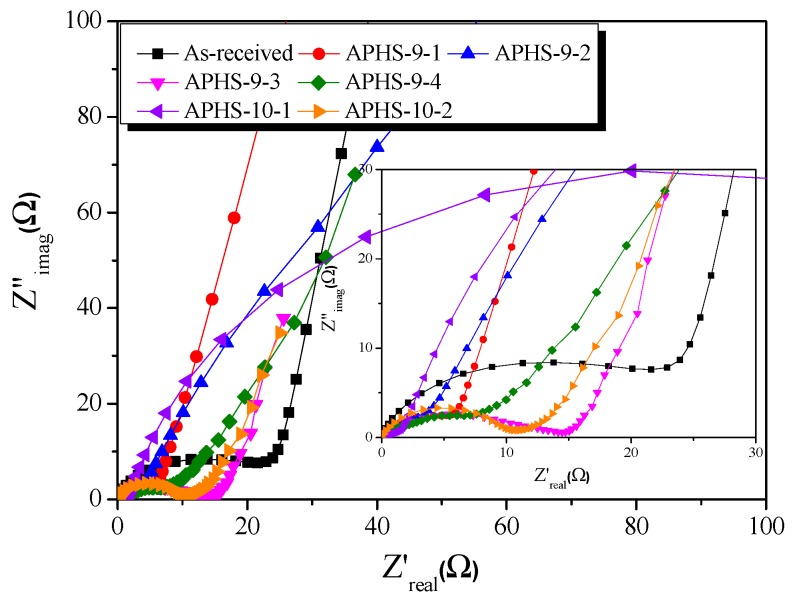
Nyquist plots of polymer-based hard carbon activated under various steam activation conditions.

Impedance spectroscopy, which distinguishes the resistance and capacitance of devices, was used to perform a comprehensive analysis of the EDLC cells. Figure 9 shows corresponding impedance plots for an organic (1M TEABF_4_/PC) electrolyte. The frequency was swept from 10 mHz to 300 kHz. Each curve presents a depressed semicircle in the middle (high-frequency region) and a nearly perpendicular line in a low-frequency region. In general, the impedance spectrum of EDLCs consists of three frequency-sensitive regions showing the characteristic shape of a Z″ = f(Z′) curve. The semicircle present at high frequencies is due to (i) electrode porosity and (ii) the charge transfer resistance of possible pseudo-capacity contributing to the total observed capacity. The electrolyte resistance is in series with the latter resistance. The electrolyte resistance influences not only the shape of the plot but also the value of ohmic resistance at which the vertical line cuts off the real resistance axis. The middle-frequency region, represented by the 45° line, is rather due to the frequency dependent resistance R(ω) associated with electrolyte penetration of the electrode pores.

All samples had the same sized semicircles at 900 °C activation temperature. Additionally, as the activation temperature increased, the size of the semicircle decreased. The higher the activation temperature, the larger were the diameter pores that developed. The charge transfer resistance decreased due to the easy movement of ions.

Pore shapes affect impedance behavior, specifically, the form of the impedance [34]. At 900 °C activation temperature, the morphology of the pore changes from a cylindrical shape (Type I) to a jar shape (Type IV) as the activation time increases. Moreover, the pore shape changes from wedge shape (Type V) to jar shape with increasing activation time at 1000 °C. At 900 °C, activation starts with pores of cylindrical form because of the slow oxidation of the graphite crystals, but at 1000 °C activation, because the oxidation of graphite crystals occurs rapidly, they develop from wedge-shaped pores. In the case of physical activation, small crystals inside the activated carbon are oxidized to form pores. Therefore, the pores of activated carbon are narrow at the entrance, and many additional pores develop inside. Finally, the pore shape of the activated carbon becomes a jar shape.

As the activation time increases, the pores develop toward the inside of the activated carbon. Therefore, as the activation time increases, the ions must move deep within the activated carbon for adsorption. As the electrolyte ion movement time increases, resistance is generated, and thereby, the slope of the straight line decreases.

## 4. Conclusions

In this work, a polymeric precursor was modified using variations of steam activation methods to prepare APHSs with high specific surface area and a mesopore-rich structure. The specific surface area and specific capacitance of the physical APHS samples was up to 2240 m^2^/g and 136 F/g, respectively.

The activation condition (time and temperature) affected the pore structure of APHS. During the initial increase of the activation time, micropores with narrow PSD were formed by oxidation of amorphous areas or small crystallites. As the activation time increased, the oxidation of crystallites increased, leading to increased specific surface areas and mesopore volume. As the activation time increased further, the oxidation of crystallites increased, leading to increased micropore and mesopore volume. With additional extension of the activation time, the pores that initially developed started to deepen, enlarge, and perhaps merge, resulting in the observed increase in the average pore width. When the activation temperature increased from 900 to 1000 °C, the oxidation rate of APHS was found to increase about twice. As a result, at the activation temperature of 1000 °C, APHS exhibited broad PSD curves and wide pore diameter.

The specific capacitance was significantly dependent on the pore size distribution according to activation conditions. It was confirmed that the correlation between the specific capacitance in 1M TEABF4/PC and the pore characteristics of the APHS was determined by pores of diameter 1.5–2.5 nm. The specific capacitance of APHS-9-4 was higher than that of APHS-10-2, which had the highest specific surface area and mesopore volume. These results suggest that the pore structure of APHS-9-4 is better optimized than is the pore structure of APHS-10-2 in 1M TEABF_4_/PC. In conclusion, APHS, created using the physical activation method, exhibited better specific capacitance than did MSP20 created using the chemical activation method.

## Figures and Tables

**Table 1:** Textural properties of polymer-based hard carbon activated under various steam activation conditions.

Sample	Activation Conditions	Yield ^2^ (%)	S_BET_^3^ (m^2^/g)	V_Total_^4^ (cm^3^/g)	V_Meso_^5^ (cm^3^/g)	V_Micro_^6^ (cm^3^/g)	C_g_^7^ (F/g)
**As-received**	-	100	50	0.03	0.01	0.02	0.8
**APHS-9-1** ^1^	900 °C × 10 min	60	1170	0.48	0.02	0.46	0.8
**APHS-9-2**	900 °C × 20 min	34	1900	0.84	0.16	0.68	73.3
**APHS-9-3**	900 °C × 30 min	33	2030	0.91	0.17	0.74	115.8
**APHS-9-4**	900 °C × 40 min	17	2240	1.16	0.38	0.78	136.2
**APHS-10-1**	1000 °C × 10 min	48	1240	0.53	0.06	0.47	26.4
**APHS-10-2**	1000 °C × 20 min	15	2410	1.22	0.47	0.75	108.2
**YP-50F**	Steam activation	-	1720	0.79	0.15	0.64	91.8
**MSP-20**	KOH activation	-	2260	0.97	0.11	0.86	125

^1^ APHS: polymer-based hard carbon activated using steam activation. ^2^ Yield: $\frac{Weight\;of\;activated\;sample}{\;Weight\;of\;hard\;carbon\;inputted}\times 100$. ^3^ S_BET_: Specific surface area by the Brunauer-Emmett-Teller (BET) method. ^4^ V_Total_: Total pore volume; BET method. ^5^ V_Meso_: Mesopore volume by the Barrett-Joyner-Halenda (BJH) method. ^6^ V_Micro_: Micropore volume; V_Total_ − V_Meso_. ^7^ C_g_: Specific capacitance.
